# Comparison of adhesion of thawed and cultured synovial mesenchymal stem cells to the porcine meniscus and the relevance of cell surface microspikes

**DOI:** 10.1186/s12860-022-00456-z

**Published:** 2022-12-12

**Authors:** Shunichi Fujii, Kentaro Endo, Nobutake Ozeki, Yuriko Sakamaki, Yuji Kohno, Mitsuru Mizuno, Hisako Katano, Kunikazu Tsuji, Hideyuki Koga, Ichiro Sekiya

**Affiliations:** 1 Center for Stem Cell and Regenerative Medicine, Tokyo Medical and Dental University (TMDU), 1-5-45 Yushima, Bunkyo-ku, Tokyo, 113-8510 Japan; 2 Research Core, Tokyo Medical and Dental University (TMDU), Tokyo, Japan; 3 Department of Cartilage Regeneration, Tokyo Medical and Dental University (TMDU), Tokyo, Japan; 4 Department of Joint Surgery and Sports Medicine, Graduate School of Medical and Dental Sciences, Tokyo Medical and Dental University (TMDU), Tokyo, Japan

**Keywords:** Mesenchymal stem cell, Synovium, Meniscus, Thawed cell, Cryopreservation, Adhesion, Microspike, Scanning electron microscopy

## Abstract

**Background:**

Placement of a cultured synovial mesenchymal stem cell (MSC) suspension on a repaired meniscus for 10 min accelerated meniscus repair. Upon placement of the MSC suspension on the meniscus, microspikes projecting from the MSC surface trap meniscus fibers and promote MSC adhesion. Thawed cryopreserved MSCs are preferred materials for meniscus repair, as they can be transplanted without additional culture. However, the adhesion ability of thawed cryopreserved MSCs is unknown. Here, we compared the proportion of cultured versus thawed MSCs adhering to a porcine meniscus immediately and 10 min after placement. We also investigated the relationship between adhesion and the number of microspikes on the synovial MSCs.

**Methods:**

Synovial MSCs were prepared from the knees of four donors with osteoarthritis. The “cultured MSCs” were thawed MSCs that were re-cultured and suspended in PBS for transplantation. A similarly prepared suspension was cryopreserved, thawed again, suspended in PBS, and used without further culture as the “thawed MSCs.” MSCs with at least three microspikes in SEM images were defined as microspike-positive MSCs. Porcine meniscus surfaces were abraded, cut into a cylindrical shape, and treated with MSC suspension. Non-adherent cells were counted immediately and again 10 min after placement to calculate the adhesion proportion.

**Results:**

The proportion of microspike-positive MSCs was significantly higher in thawed (53 ± 3%) than in cultured (28 ± 5%) MSC suspensions. MSC adhesion to the meniscus was significantly better for the thawed than for the cultured MSC suspensions immediately after placement on the meniscus, but no differences were detected after 10 min. The proportion of MSCs with microspikes in the cell suspension was significantly correlated with the proportion of adhered MSCs immediately after the placement, but not 10 min later. Addition of FBS to the cryopreservation medium promoted a concentration-dependent increase in the proportion of microspike-positive cells.

**Conclusions:**

Thawed MSCs adhered better than cultured MSCs immediately after placement, but adhesion was similar for both MSC preparations after 10 min. Immediately after placement, the presence of microspikes was associated with better adhesion of synovial MSCs to the meniscus.

## Introduction

The meniscus is a fibrocartilaginous tissue in the knee and plays critical roles in the stability, lubrication, and load-bearing distribution of the knee joint [1]. Because of these important functions, symptomatic meniscus tears are a common clinical problem [2]. However, the healing ability of the meniscus is limited, so meniscus tears are typically treated by a partial meniscectomy, rather than surgical repair, in approximately 70% of injury cases [3]. Unfortunately, meniscectomies often accelerate knee osteoarthritis [4]; therefore, alternative treatments are needed for meniscus repair.

One promising new protocol for healing meniscus tears is the transplantation of mesenchymal stem cells (MSCs). MSCs derived from the knee synovium (i.e., synovial MSCs) have a particularly high proliferative and chondrogenic potential [5], making them highly attractive for meniscus healing compared to MSCs derived from other tissues. The placement of a suspension of cultured synovial MSCs onto a surgically repaired pig meniscus for 10 min was shown to accelerate meniscus healing [6]. This same treatment was also effective in human patients with meniscus tears who would otherwise have undergone meniscectomy [7].

The details of the process by which synovial MSCs adhere to the meniscus surface remained obscure until a recent in vitro study demonstrated that 33% of cultured human synovial MSCs would adhere to a porcine meniscus within 10 min of placement. Even immediately after placement, 28% of the MSCs had already adhered to the meniscus, apparently via microspikes present on the MSC surface. These microspikes, which are thin, short processes that project from the MSC surface, became trapped in the fibers of the meniscus surface [8]. Therefore, the MSC microspikes are possibly responsible for the initial attachment of the MSCs to the meniscus.

These previous studies used freshly cultured synovial MSCs. However, for clinical applications, the use of cryopreserved MSCs is favored, as these cells can simply be thawed and used without the need for any extra culture step, thereby allowing convenient adjustment of the transplantation schedule as required. One unknown factor is whether thawed cryopreserved MSCs will have the same capacity as freshly cultured MSCs to adhere to the meniscus surface. The purpose of the present study was to compare the proportion of thawed MSCs that adhere to the meniscus immediately and 10 min after placement versus the proportion of cultured MSCs that adhere at the same time points. A second goal was to investigate the relationship between the proportion of adhering cells and the presence of microspikes on synovial MSCs.

## Methods

### Isolation of human synovial MSCs

This study was approved by the Medical Research Ethics Committee of Tokyo Medical and Dental University, and informed consent was obtained from all study subjects. Human synovium was harvested from the knees of four donors during total knee arthroplasty operations performed to treat osteoarthritis. The synovium was minced and digested in a solution of 3 mg/mL collagenase (Sigma-Aldrich Japan, Tokyo, Japan) at 37 °C for 3 h, and the digested cells were filtered through a 70 μm cell strainer (Greiner Bio-One GmbH, Frick-enhausen, Germany). The obtained nucleated cells were cultured in a growth medium consisting of α-MEM (Thermo Fisher Scientific, Rockford, IL, USA), 1% antibiotic-antimycotic (Thermo Fisher Scientific), and 10% fetal bovine serum (FBS, Thermo Fisher Scientific) under 5% CO_2_ at 37 °C. After 14 days, the human synovial MSCs were detached with 0.25% trypsin and 1 mM EDTA, harvested, and cryopreserved at − 80 °C for future use at a concentration of 10^6^ cells/mL in a freezing vessel (BICELL, Japan Freezer, Tokyo, Japan). The cryopreservation medium consisted of 95% growth medium plus 5% dimethyl sulfoxide (DMSO, Fujifilm Wako Pure Chemical, Osaka, Japan). For colony formation assays, 100 cells, at 1.67 cells/cm^2^ in a 60 cm^2^ dish (Nunc EasYDish, Thermo Fisher Scientific) were cultured for 14 days and then stained with crystal violet (Fujifilm Wako Pure Chemical).

### Differentiation assays

Chondrogenesis was examined by suspending 2.5 × 10^5^ human synovial MSCs in 0.5 mL chondrogenic induction medium consisting of DMEM (Thermo Fisher Scientific) supplemented with 10 ng/mL transforming growth factor-β3 (Miltenyi Biotec, Bergisch Gladbach, Germany), 500 ng/mL bone morphogenetic protein 2 (BMP-2, Medtronic, Minneapolis, MN, USA), 40 μg/mL proline, 100 nM dexamethasone (Fujifilm Wako Pure Chemical), 100 μg/mL pyruvate, 50 μg/mL ascorbate-2-phosphate (Fujifilm Wako Pure Chemical), and 1% ITS Premix (Becton Dickinson [BD], NJ, USA). The cells were pelleted by centrifugation at 500×g for 10 min and then cultured for 21 days. The pellets were sectioned and stained with safranin O (Fujifilm Wako Pure Chemical).

For adipogenesis, 100 synovial MSCs were cultured in a 60 cm^2^ dish for 14 days in growth medium to produce cell colonies. The adherent cells were cultured for a further 21 days in an adipogenic induction medium consisting of α-MEM supplemented with 100 nM dexamethasone, 0.5 mM isobutylmethylxanthine (Sigma-Aldrich), and 50 mM indomethacin (Fujifilm Wako Pure Chemical). The generated adipocytes were stained with oil red O (Muto Pure Chemicals, Tokyo, Japan).

For calcification, 100 synovial MSCs were cultured in a 60 cm^2^ dish for 14 days in growth medium to produce cell colonies. The adherent cells were cultured for a further 21 days in calcification medium consisting of growth medium supplemented with 50 μg/mL ascorbate-2-phosphate, 10 nM dexamethasone, and 10 mM β-glycerophosphate (Sigma-Aldrich). Calcification was assessed by alizarin red staining (Merck Millipore, Billerica, MA, USA).

### Flow cytometry

Human synovial MSCs were detached with TrypLE (Thermo Fisher Scientific) and suspended in FACS buffer (0.2% FBS and 5 mM EDTA [Thermo Fisher Scientific] in phosphate-buffered saline [PBS]) at a density of 5 × 10^5^ cells/mL. Thawed MSCs were used immediately after thawing, with no further culture. The cells were stained for 30 min with the following antibodies: CD44 (PE-Cy7), CD45 (APC-H7), CD73 (V450), CD90 (PE), and CD105 (APC) (all from BD). Cell fluorescence was evaluated using a FACS Verse instrument (BD). The data were analyzed using FlowJo software (Tree Star Software, CA, USA).

### Preparation of cultured and thawed human synovial MSCs

**Fig. 1:**
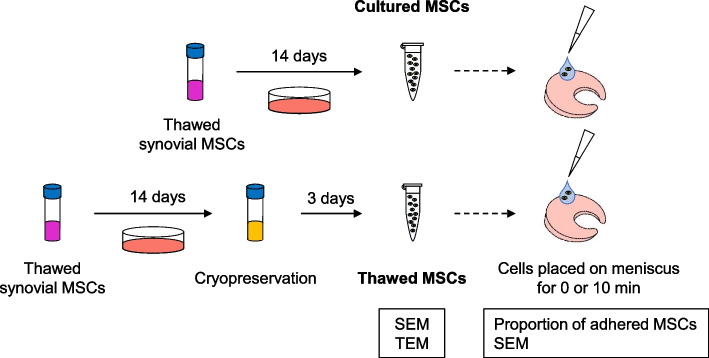
Schematic of the study design. For cultured MSCs, the thawed cells were cultured for 14 days, and then suspended in PBS. For thawed MSCs, the thawed cells were cultured for 14 days, cryopreserved in 95% FBS and 5% DMSO for 3 days, and then suspended in PBS without further culturing. The cultured and thawed MSCs were observed by SEM and TEM. The MSCs were placed on porcine menisci, and the proportion of adhering cells and the cell SEM morphology were analyzed immediately and 10 min after MSC placement

For cultured MSCs, the cryopreserved cells were thawed (ThawSTAR, Astero Bio, Menlo Park CA, USA), cultured for 14 days, and suspended in PBS for use in transplantation. For thawed MSCs, the cells were similarly thawed and cultured for 14 days, but they were subsequently cryopreserved at − 80 °C in 95% FBS and 5% DMSO at a density of 10^7^ cells/mL [9] for 3 days. The cells were then thawed and suspended in PBS for use in transplantation (Fig. 1). The effect of FBS concentration on microspikes was evaluated by cryopreserving the cells were in the following solutions: (i) α-MEM supplemented with 10% FBS and 5% DMSO, (ii) α-MEM supplemented with 50% FBS and 5% DMSO, (iii) 95% FBS and 5% DMSO.

### Adhesion of human synovial MSCs to procine menisci

The surface of the menisci excised from fresh porcine knees (Shibaura Zoki Co., Ltd., Tokyo, Japan) was abraded to reproduce a degenerative meniscus. Each meniscus was cut into a cylindrical shape 12 mm in diameter. A cell suspension containing 10^6^ cultured or thawed MSCs in 100 μL PBS (10^7^ cells/mL) was placed on the meniscus. Either immediately after placement or 10 min later, the meniscus was washed with 1 mL PBS. The proportion of MSCs adhering to the meniscus was then calculated by counting the numbers of non-adherent cells in the washes [8].

### Scanning electron microscopy

Cells and menisci were fixed in 2.5% glutaraldehyde in 0.1 M phosphate buffer (PB) for 2 h and washed overnight in 0.1 M PB at 4 °C. The specimens were then post-fixed with 1% osmium tetroxide (OsO_4_) in 0.1 M PB for 2 h at 4 °C and dehydrated in graded ethanol solutions. After exchanging with 3-methyl butyl acetate and critical point drying, the specimens were coated with platinum, and the meniscus surface was observed by SEM (S-4500; Hitachi Ltd., Tokyo, Japan). For quantification of microspike-positive cells, 50 cells were randomly selected per examination. The selected cells were classified by the presence or absence of microspikes. Microspike-positive cells were defined as cells that contained at least three microspikes [8].

### Transmission electron microscopy (TEM)

Cells were fixed in 2.5% glutaraldehyde in 0.1 M PB for 2 h, washed with 0.1 M PB, and post-fixed in 1% OsO_4_ in 0.1 M PB for 2 h. After washing with PB, cells were resuspended in 2% gelatin (Sigma-Aldrich) and pelleted again. Microcentrifuge tubes were plunged into ice-cold water to quickly solidify the gelatin with the cells. The tip of the tube was cut open, and the cell pellets were cut into 1 mm^3^ blocks, dehydrated in a graded series of ethanol, and embedded in Epon 812. Ultrathin sections 70 nm thick were collected on copper grids and double-stained with uranyl acetate and lead citrate. The sections were examined by TEM (JEM-1400Flash, JEOL, Tokyo, Japan).

### Statistical analysis

The proportions of adhered cultured versus thawed MSCs were compared using Student’s paired *t*-test. The correlation between the proportion of cells with microspikes in the cell suspension and the proportion of adhered cells was statistically evaluated with Pearson’s product-moment correlation. The effect of FBS concentration on microspikes was evaluated using Tukey’s test. Data were expressed as the average ± standard deviation (SD). *P* values < 0.05 were considered statistically significant. All statistical analyses were performed using GraphPad Prism 6 (GraphPad Software, CA, USA).

## Results

### Characteristics of synovium-derived cells as MSCs

**Fig. 2:**
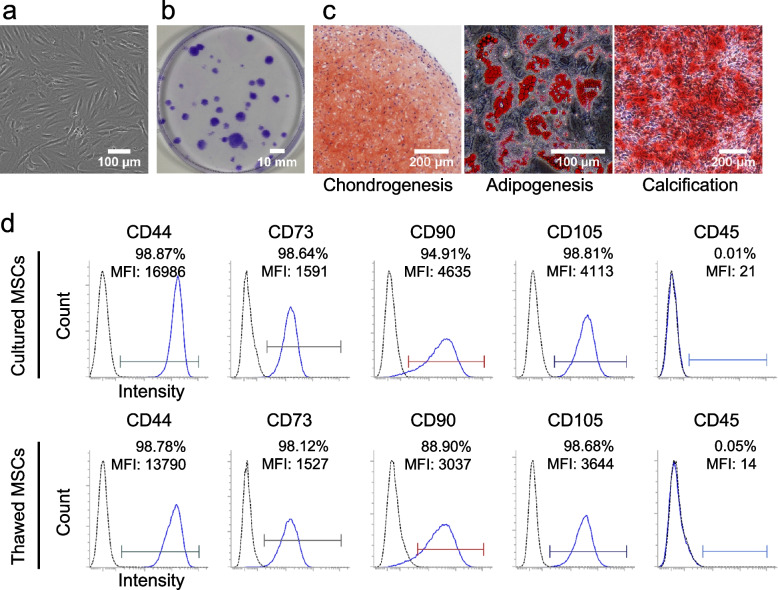
Properties of human synovial MSCs. **a** Cell morphology. **b** Colony morphology. **c** Multidifferentiation potential. **d** Surface markers of cultured and thawed MSCs. The dotted line indicates the isotype control. MFI, mean fluorescent intensity

The human synovium-derived cells showed a spindle-shaped morphology (Fig. 2a) and colony-forming ability (Fig. 2b). After chondrogenic, adipogenic, and calcification induction, they stained positive for safranin O, oil red O, and alizarin red, respectively (Fig. 2c). Both cultured and thawed MSCs expressed CD44, 73, 90, and 105, but not CD45 (Fig. 2d). All these characteristics meet the criteria that define MSCs [10] and indicated that the synovium-derived cells we prepared were MSCs.

### Morphology of cultured and thawed MSCs in suspension

**Fig. 3:**
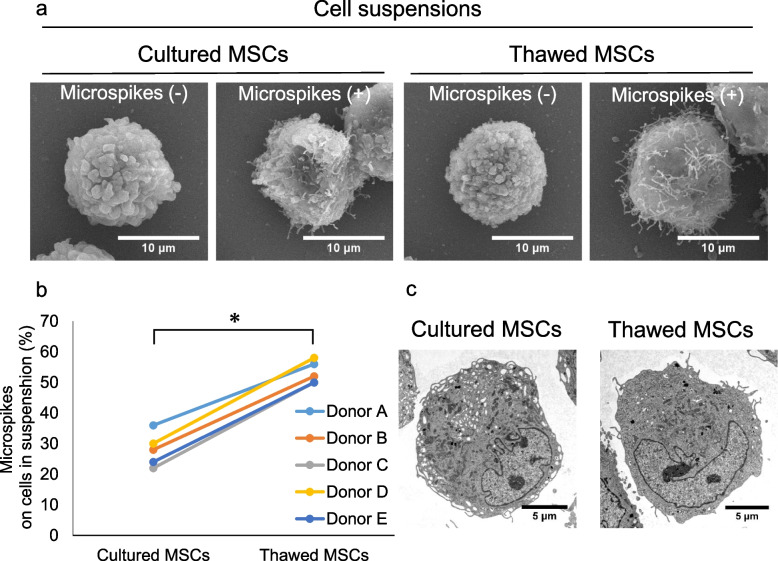
Morphology of cultured and thawed synovial MSCs in suspension. **a** SEM images. **b** The proportion of cells with microspikes. Fifty cells were examined once per donor, and the results from five donors are shown independently. The proportion of MSCs with microspikes was 28 ± 5% in the cultured MSC suspension and 53 ± 3% in the thawed MSC suspension.*; *p* < 0.05 by paired t-test. **c** Representative TEM images

SEM observation demonstrated that both cultured MSCs and thawed MSCs in suspension could be divided into two types: those with and those without microspikes (Fig. 3a). The proportion of MSCs with microspikes was significantly higher in the thawed MSC suspension (53 ± 3%) than in the cultured MSC suspension (28 ± 5%) (Fig. 3b). TEM observation showed that both cultured and thawed MSCs had similar well-developed organelles (Fig. 3c).

### MSC adhesion 10 min after placement on the meniscus

SEM observations revealed a higher number of cultured MSCs adhering to the meniscus 10 min after placement than immediately after placement (Fig. 5a). By contrast, no difference in adhesion was noted for the thawed MSCs placed either immediately or after 10 min (Fig. 5a). Quantitative analysis showed no significant difference in the proportion of adhered cells between the cultured and the thawed MSCs (Fig. 5b). No correlation was detected between the proportion of adhered cells at 10 min after placement of the MSCs on the meniscus and the proportion of MSCs with microspikes in the cell suspension (Fig. 5c).

### MSC adhesion immediately after placement on the meniscus

**Fig. 4:**
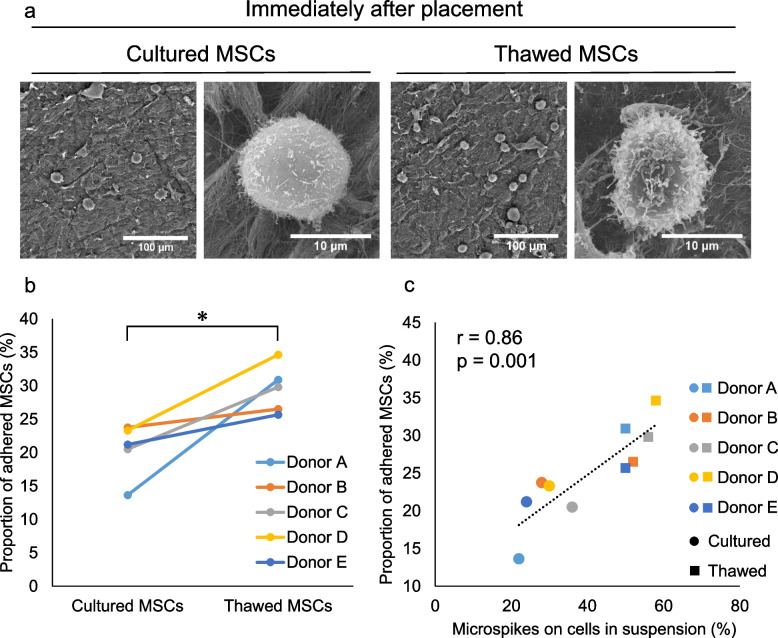
Morphology and adhesion proportion of human synovial MSCs immediately after placement on the porcine meniscus. **a** SEM images. **b** Proportion of cells adhered to the meniscus. Six cylindrical menisci were examined once per donor, and the results from five donors are shown independently. The proportion of adhering cells was 20 ± 4% for the cultured MSCs and 30 ± 3% for the thawed MSCs. *; *p* < 0.05 by paired t-test. **c** Correlation between the proportion of adhered cells and the proportion of cells with microspikes in suspension

SEM observation indicated that the number of MSCs adhering to the meniscus immediately after placement was higher for the thawed MSCs than for the cultured MSCs (Fig. 4a). Quantitative analysis confirmed that the proportion of adhering cells was significantly higher for the thawed MSCs than for the cultured MSCs (Fig. 4b). A significant correlation was detected between the proportion of adhering cells immediately after placement of the MSCs on the meniscus and the proportion of MSCs with microspikes in the cell suspension (Fig. 4c).

**Fig. 5:**
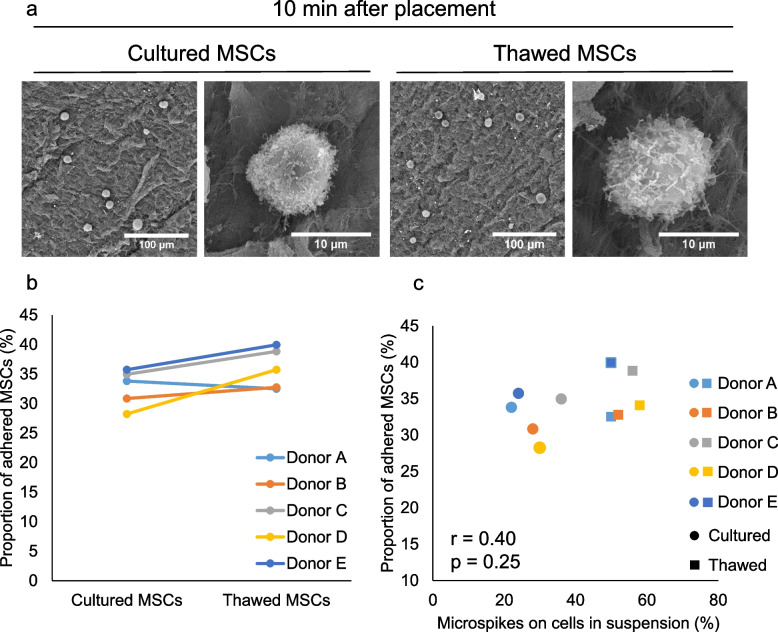
Morphology and adhesion proportion of human synovial MSCs 10 min after placement on the porcine meniscus. **a** SEM images. **b** Proportion of cells adhered to the meniscus. Six cylindrical menisci were examined once per donor, and the results from five donors are shown independently. The proportion of adhering cells was 33 ± 3% for the cultured MSCs and 36 ± 3% for the thawed MSCs. *;*p* < 0.05 by paired t-test. **c** Association between the proportion of adhered cells and the proportion of cells with microspikes in suspension

**Fig. 6:**
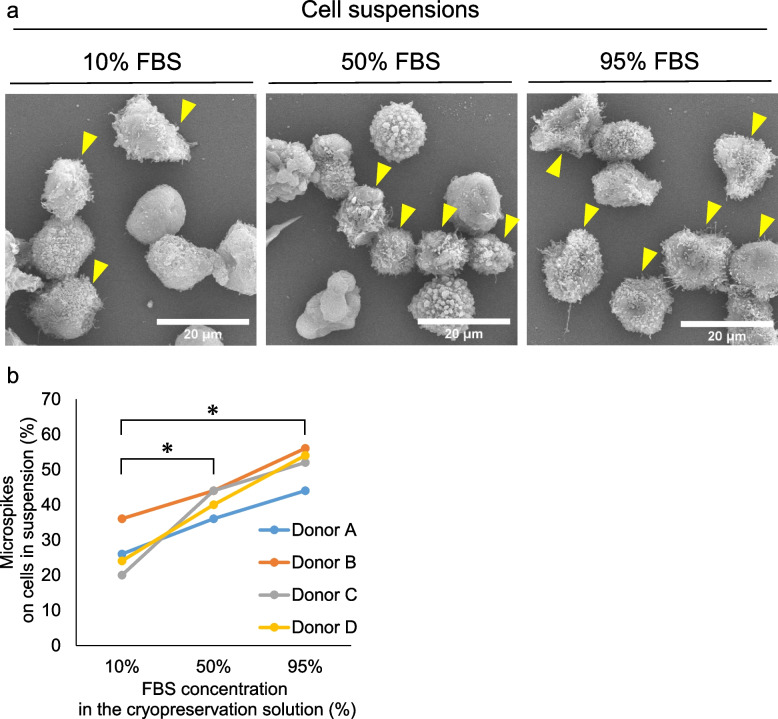
Morphology of MSCs cryopreserved in solutions containing with different concentrations of fetal bovine serum (FBS). **a** SEM images. Cells with microspikes are marked with arrowheads. **b** The proportion of cells with microspikes. Fifty cells were examined once per donor, and the results from four donors are shown independently. The proportion of MSCs with microspikes was 27 ± 6% in the 10% FBS, 52 ± 5% in the 50% FBS, and 52 ± 5% in the 95% FBS. *; *p* < 0.05 by Tukey’s test

### The effect of FBS concentration on microspikes

The effect of the FBS concentration in the cryopreservation solution on the formation of microspikes was evaluated in MSCs crypreserved in solution containing 10, 50, and 95% FBS. The addition of FBS caused a concentration-dependent increase in the expression of MSC microspikes (Fig. 6a, b). The proportion of cells with microspikes was significantly greater in medium contatining 50% FBS (41 ± 3%) or 95% FBS (52 ± 5%) than 10% FBS (27 ± 6%).

## Discussion

We compared the proportion of cells adhering to the meniscus between cultured and thawed MSCs immediately or 10 min after placement to investigate the relationship between the proportions of adhering cells and the presence of microspikes on synovial MSCs. The proportion of cells with microspikes was 28 ± 7% for the cultured MSC suspensions and 52 ± 3% for the thawed MSC suspensions, and the difference was statistically significant. The proportion of MSCs adhering to the meniscus was significantly higher for the thawed MSCs than for the cultured MSCs immediately after the placement, but no differences were detected at 10 min after placement. The proportion of adhering cells was significantly correlated with the proportion of MSCs with microspikes in the cell suspension immediately after placement, but not after 10 min.

The proportion of microspike-positive cells was significantly higher for the thawed MSCs than for the cultured MSCs. Our results differ from those presented in the systematic review by Bahsoun et al., who reported no difference in morphology between thawed and cultured MSCs [11]. This discrepancy might reflect our use of SEM for our analysis, whereas many other studies have used light microscopy. The resolving power of light microscopy is limited, so SEM may have had an advantage in revealing fine details, such as microspikes. In support of this, an SEM study by James et al. demonstrated that thawed human adipose-derived MSCs were more stellate than cultured cells [12]. We also used a cryopreservation solution containing 95% FBS. Various concentrations of FBS have been used for MSC cryopreservation, but 95% is the highest [11]. FBS contains several growth factors and proteins that promote cell adhesion [13, 14]. Kim et al. reported that FBS altered the cytoskeletal structure and cell morphology of human umbilical cord-derived MSCs and promoted ITGB1-mediated cell adhesion [15]. Therefore, we also examined the effect of the FBS concentration in the cryopreservation solution on microspike formation. Quantitative analysis using SEM revealed that FBS induced a concentration-dependent increase in the proportion of MSCs with microspikes. Therefore, the concentration of FBS could possibly modulate cell morphology and affect the development of microspikes during the freeze-thaw process in human synovial MSCs.

The proportion of adhered MSCs was higher immediately after placement for the thawed MSCs than for the cultured MSCs. In our previous study, cultured human synovial MSCs with microspikes were most frequently observed immediately after placement on the meniscus, and SEM analysis demonstrated that microspikes could be physically trapped by meniscus fibers [8]. In the present study, more cells with microspikes were observed in the thawed MSC suspensions, suggesting that those cells were more likely to become trapped in the meniscal fibers, thereby increasing the proportion of adhering cells. A statistically significant correlation was also observed between the proportion of MSCs with microspikes in the cell suspension and the proportion of cells adhering immediately after the placement of the MSC suspension. These results demonstrate that microspikes on the surface of human synovial MSCs play an important role in the initial attachment of MSCs to the meniscus.

At 10 min after the placement on the meniscus, the proportion of adhering cells increased for the cultured MSCs, but did not change for the thawed MSCs. Consequently, no significant difference was observed in the proportion of adhered cells between the cultured and thawed MSC preparations. Many studies have shown that thawed MSCs have a lower adherence potential [12, 16–18]. Furthermore, Tan et al. reported that thawed MSCs began to show an increase in both early and late apoptotic cells immediately after preparation [19]. Although we did not evaluate MSC viability and apoptosis in our cultured and thawed MSC preparations, an increase in apoptotic cells in the thawed MSC preparation could have prevented an increase in the proportion of adhering cells. Nevertheless, our results demonstrated that the number of cells that adhered to the meniscus did not differ between the cultured and thawed MSC preparations, indicating that thawed MSCs can be substituted for cultured MSCs for the treatment of meniscus tears.

Increasing the initial adhesion of MSCs is necessary to improve the outcome of meniscus repair by MSC transplantation [7]. One strategy to achieve this is to increase the proportion of MSCs with microspikes [8]. In this study, we analyzed the effect of cryopreservation and the concentration of FBS in the cryopreservation solution on microspikes. In addition, composition of cryopreservation solution other than FBS, speed of thawing, and surface antigens of MSCs may also affect the proportion of cells with microspikes. This method will lead to an increase in the proportion of MSCs with microspikes from these perspectives, which will result in improved results in clinical applications.

This study had a few limitations. One was that we only examined the proportion of adhering cells up to 10 minutes after the placement, and this time interval is not sufficient to determine the definitive ability of thawed MSCs to adhere to the meniscus. Another limitation is that we cryopreserved the MSCs for only 3 days. In clinical settings, the cryopreservation period is usually longer. A third limitation is that we focused only on microspikes in our SEM analysis. Morphological changes in the pseudopodia may also be related to MSC adhesion, but these structures were difficult to observe at 10 min after cell placement.

## Conclusions

Thawed MSCs showed better adhesion than cultured MSCs immediately after placement on porcine menisci and showed equivalent adhesion at 10 min after placement. Immediately after the placement, the presence of microspikes was associated with better adhesion of synovial MSCs to the meniscus. Higher concentrations of FBS in the cryopreservation solution increased the expression of microspikes.

## Data Availability

The datasets used and/or analyzed during the current study are available from the corresponding author on reasonable request.

## Abbreviations

DMSO: Dimethyl sulfoxide
FBS: Fetal bovine serum
MSCs: Mesenchymal stem cells
PBS: Phosphate-buffered saline
SEM: Scanning electron microscopy
PB: Phosphate buffer
OsO_4_: Osmium tetroxide
TEM: Transmission electron microscopy
SD: Standard deviation
